# Public antibodies: convergent signatures in human humoral immunity against pathogens

**DOI:** 10.1128/mbio.02247-24

**Published:** 2025-04-16

**Authors:** Vishal N. Rao, Camila H. Coelho

**Affiliations:** 1 Department of Microbiology, Icahn School of Medicine at Mount Sinai 5925, New York, USA; 2 Center for Vaccine Research and Pandemic Preparedness, Icahn School of Medicine at Mount Sinai 5925, New York, USA; 3 Graduate School of Biomedical Sciences, Icahn School of Medicine at Mount Sinai 5925, New York, USA; 4 Precision Immunology Institute, Icahn School of Medicine at Mount Sinai 5925, New York, USA; The Ohio State University, Columbus, Ohio, USA

**Keywords:** public antibodies, human pathogens, antibody repertoire, V(D)J recombination, somatic hypermutation

## Abstract

The human humoral immune system has evolved to recognize a vast array of pathogenic threats. This ability is primarily driven by the immense diversity of antibodies generated by gene rearrangement during B cell development. However, different people often produce strikingly similar antibodies when exposed to the same antigen—known as public antibodies. Public antibodies not only reflect the immune system’s ability to consistently select for optimal B cells but can also serve as signatures of the humoral responses triggered by infection and vaccination. In this Minireview, we examine and compare public antibody identification methods, including the identification criteria used based on V(D)J gene usage and similarity in the complementarity-determining region three sequences, and explore the molecular features of public antibodies elicited against common pathogens, including viruses, protozoa, and bacteria. Finally, we discuss the evolutionary significance and potential applications of public antibodies in informing the design of germline-targeting vaccines, predicting escape mutations in emerging viruses, and providing insights into the process of affinity maturation. The ongoing discovery of public antibodies in response to emerging pathogens holds the potential to improve pandemic preparedness, accelerate vaccine design efforts, and deepen our understanding of human B cell biology.

## INTRODUCTION

The human humoral immune response is mediated by antibodies, which are secreted in response to antigen exposure and are critical to protect against infection (1–3). Antibodies display enormous diversity that enables the recognition of a wide variety of antigens (4, 5). This diversity originates early in life during B cell development when multiple combinations of V(D)J genes rearrange to encode the variable regions of antibody heavy and light chains (6–9). The variable region consists of three complementarity-determining regions (CDRs), which primarily contact the antigen, and four framework regions (FWRs) that maintain the variable region’s structural integrity (10, 11). Antibody diversity takes two forms: (i) combinatorial diversity, which refers to the numerous combinations of V, D, and J gene segments that recombine to form a single VDJ exon (12–15) and (ii) junctional diversity, referring to the insertion or deletion of nucleotides at the V-J/D-J/V-J junctions (16, 17). The junctional region mainly contains the CDR3, which greatly influences antigen interactions (18–20), making junctional diversity particularly important for antigen specificity.

Despite the possibility for vast diversity, humans tend to produce similar antibodies against specific epitopes of given antigens, which are known as "public antibodies” (21–23). This phenomenon of convergence in antibody responses in multiple individuals warrants the need to study the origin and applicability of public antibody responses in mounting effective humoral responses against emerging pathogens, either through natural infection or vaccination. In this Minireview, we summarize the key published findings on public antibodies induced by pathogen exposure in humans. We also review the tools used to identify these antibodies and emphasize the potential applications and benefits of harnessing public antibodies within the context of human biology.

## TOOLS TO IDENTIFY HUMAN PUBLIC ANTIBODIES

Public antibodies are typically identified by sequencing their variable regions. Antibody sequences can be determined from (i) B cells, which express mRNA transcripts encoding either antibodies or the B cell receptor (BCR) (24–27) or (ii) secreted antibodies isolated from the serum after infection or vaccination (28, 29). In this section, we describe the technical approaches used to identify public antibodies by analyzing BCRs and secreted antibodies.

### High-throughput single-cell V(D)J sequencing

V(D)J sequencing has advanced from the low-throughput sequencing of heavy chains from bulk B cells (30–32) to high-throughput, next-generation paired heavy and light chain V(D)J sequencing of single B cells (33–37), which greatly facilitates antibody sequence reconstruction and *in vitro* monoclonal antibody expression (37–42). Single-cell V(D)J sequencing can also be applied to B cell subpopulations such as plasmablasts, plasma cells, germinal center B cells, and naïve B cells from various anatomical compartments (e.g., the peripheral blood, lymphoid tissues, and bone marrow), offering mechanistic insights into antibody origins (26, 43). V(D)J sequencing can also be coupled with tools like B cell tetramers that enable the capture of rare antigen-specific B cells (44–47) or techniques like LIBRA-seq which involves simultaneous capture of multiple antigen-specific B cells by using unique oligonucleotide barcodes that are conjugated to each antigen of interest (48, 49). As a result, advances in single-cell sequencing have not only facilitated the discovery of novel antibodies targeting human pathogens but also enabled the identification of public antibodies within human cohorts.

### Proteomics-based antibody sequencing

Since most serum antibodies originate from short-lived plasmablasts or plasma cells in the bone marrow (50), the peripheral blood B cell repertoire does not entirely reflect the serum antibody repertoire (29, 51). Proteomics-based antibody sequencing, also referred to as Ab-seq, involves isolating antigen-specific antibodies from the sera or plasma, followed by proteolytic digestion to peptides, which are analyzed by liquid chromatography coupled to mass spectrometry. They are then mapped to the sequences of germline V(D)J genes, and CDR3s are reconstructed using informatics-based approaches (52–55). This proteomics approach also enables the study of post-translational modifications, which can impact an antibody’s binding, half-life, and effector functions (56). Voss and colleagues identified severe acute respiratory syndrome coronavirus 2 (SARS-CoV-2) spike protein-specific public antibodies in coronavirus disease 2019 (COVID-19) convalescent sera. They found that the response was dominated by N-terminal domain (NTD)-targeting antibodies primarily encoded by immunoglobulin heavy variable 1–24 (*IGHV1-24*) gene (57). Adamson and colleagues showed that *IGHV3-7* and *IGHV5-51* are the most used heavy chain V genes in serum antibodies elicited upon influenza A/(H1N1) pdm09 vaccination (58). However, the utility of this approach to identify public antibodies is largely limited by the low resolution of the CDR3 and somatic hypermutations, and inadequate detection of clonal lineages (56), making V(D)J gene sequencing a more popular and efficient tool for discovering public antibodies in human cohorts.

## IDENTIFYING PUBLIC ANTIBODIES IN ANTIBODY REPERTOIRES

To identify public antibodies based on sequence similarity, antibody sequences are typically mapped to their germline V(D)J gene segments and assigned to their FWRs and CDRs using tools such as IgBLAST (59). Most public antibody identification criteria rely on two fundamental features: V and J gene usage, and CDR3 similarity (60–63), which assess the combinatorial and junctional diversity gained during V(D)J recombination, respectively (12, 20). These criteria and the advantages and disadvantages of defining public antibodies based on gene usage and/or CDR3 similarity are outlined here.

### Gene usage

Gene usage is typically a low-stringency criterion, which uses the presence of molecular features encoded within certain V genes that preferentially support binding to certain epitopes to identify broad classes of public antibodies. A well-known example of public antibodies defined by V gene usage are the influenza virus hemagglutinin (HA) stem-directed *IGHV1-69* antibodies (64–70) that comprise approximately 50%–60% of stem-directed antibodies (71). Another example is the class of *IGHV3-53/66* neutralizing antibodies against SARS-CoV-2 that target the receptor-binding domain (RBD) of the spike protein and compete with its binding to angiotensin-converting enzyme 2 (ACE2) on host cells (72–76). In these antibodies, a germline-encoded NY motif in CDRH1 and an SGGS motif in CDRH2 are key for RBD binding, while additional interactions from CDRH3s of different lengths, varying light chains, and somatic hypermutations improve the breadth of recognition (74, 77, 78). In addition to the conventional use of V and J genes, Wang and colleagues recently showed that inclusion of the D gene *IGHD1-26* constitutes a public antibody response specific to the S2 subunit of the SARS-CoV-2 spike protein (63). Several studies have identified light chain-based convergence in public antibodies (63, 79, 80). We recently showed that in a class of SARS-CoV-2 S2-specific public antibodies using the *IGHV4-59* and *IGKV3-20* genes, convergent SHMs in the light chain, but not the heavy chain, were enriched in this clonotype and drove affinity maturation of this public antibody class (80).

Thus, defining public antibodies based on V gene usage is highly advantageous to identify repertoire signatures that characterize B cell responses to specific antigens. Due to its limited stringency, the number of sequences required to identify public antibodies is low, enabling the identification of public antibodies in broader cohorts and databases with varying levels of sequencing depth. On the other hand, V(D)J gene usage is insufficient to completely define clonotypes (i.e., a group of B cells with unique gene rearrangements originating from a common germline precursor). Thus, there is an inherent loss in specificity when classifying public antibodies using gene usage alone. Additionally, defining public antibodies based on gene usage typically prioritizes the heavy chain. This reduces the specificity further, as recent reports revealed that the light chain is critical to identify public clones (63).

A further layer of complexity that could potentially determine the classification of public antibodies based on gene usage is the presence of allelic polymorphisms within V genes. Previous studies on antigen-antibody interactions in influenza virus, HIV-1, *Plasmodium falciparum,* and *Staphylococcus aureus* have demonstrated that single amino acid alterations caused by V gene polymorphisms, often in the CDRs, can notably affect the affinity for the cognate antigen, with certain allelic variants showing a complete loss of affinity to the antigen (69, 81–85). Incorporating V gene alleles into the classification of public antibodies could refine this classification’s specificity and facilitate the design of broadly effective vaccines for the general population.

### CDR3 similarity

CDR3 is crucial for determining antigen specificity, as it is directly involved in most antigen-antibody binding interactions (18–20). Combined with gene usage, CDR3 similarity is a high-stringency method to identify public antibodies that accounts for both combinatorial and junctional diversity. The CDR3-dependent criteria to define public antibodies involve the region’s length and sequence similarity, which are typically analyzed in combination to ensure high specificity (63, 86). The length restriction (i.e., grouping sequences based on identical CDR3 length) ensures that the length of the junctional region generated during recombination remains consistent within a class of public antibodies (16, 17). The requirement for high sequence similarity ensures functional similarity in epitope recognition (20, 63). The similarity threshold varies across studies, with 70% typically being the least stringent (87–89). More stringent thresholds typically improve the specificity of identifying public antibodies (60).

The main advantage of using CDR3 similarity to define public antibodies is its high accuracy. Wang and colleagues used an identical CDRH3 length and ≥80% CDRH3 amino acid similarity as criteria to define clusters of public antibodies from over 8,000 sequences of human antibodies against SARS-CoV-2 from 88 studies and found that antibodies grouped in the same clusters mapped to the same epitope regions (i.e., the RBD, NTD, or S2 subunit) (63), highlighting the power of these criteria.

### Limitations in identifying public antibodies

Defining public antibodies using both V gene usage and CDR3 similarities in the heavy and light chains is a robust approach that ensures that antibodies identified in different individuals are derived from similar germlines (90). However, this approach may overlook other molecular features that could influence antibody specificity. For instance, sequence motifs within CDR1 or CDR2 can contribute to antigen recognition and can be shared by different clonotypes with similar antigen-binding sites (60, 71). This suggests that while high-stringency criteria based on gene usage and CDR3 similarity can increase specificity, they may also exclude antibodies that recognize the same epitope through other shared features in the antigen-binding site. In addition, Jaffe and colleagues demonstrated the phenomenon of “light chain coherence” among public antibody clonotypes. By defining public antibodies based on heavy chain V and J gene usage, an identical CDRH3 length, and ≥90% CDRH3 amino acid similarity, they found that public antibodies from antigen-experienced and functional memory B cells tended to share the same light chains (with up to 80% coherence). In contrast, antibodies derived from naive B cells showed much lower light chain coherence (approximately 10%) (91). This indicates that heavy and light chain convergence based on gene usage and CDR3 similarity are not entirely independent of each other. Nonetheless, identifying public antibodies based on gene usage and CDR3 similarity independently in the heavy and light chains can inform key paratope features within each chain that determine binding.

**Fig 1:**
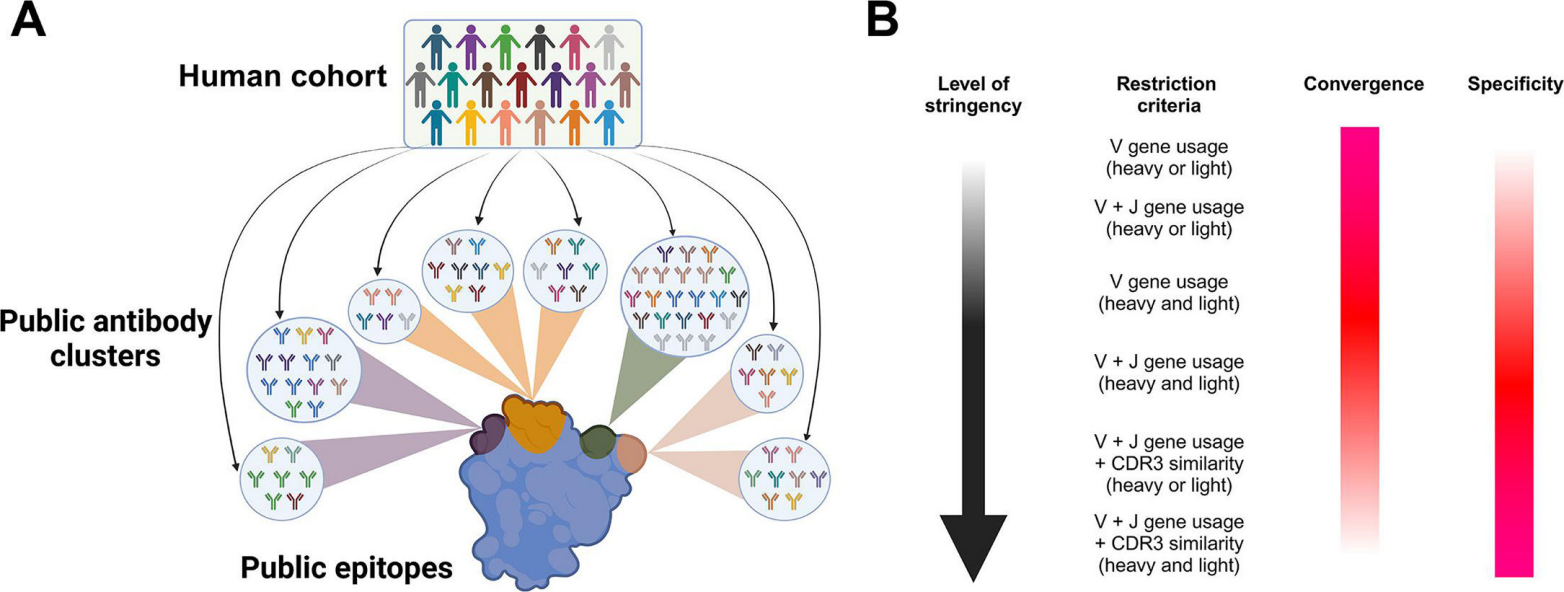
Identifying public antibodies and epitopes in human cohorts. (**A**) Public antibodies vs public epitopes. Public antibodies from different clusters, as well as non-public antibodies (not shown), can recognize the same or overlapping epitopes, termed public epitopes. (**B**) Common criteria employed to identify public antibodies from immunoglobulin variable region sequences. Increased stringency increases the specificity (i.e., the likelihood of public antibodies within a cluster recognizing the same or overlapping epitope) but could also decrease the convergence of public antibodies in a human cohort or population (i.e.*,* fewer antibodies will be clustered within each class of public antibodies).

In addition to generating public antibodies, the human antibody repertoire also tends to target convergent epitopes. Zhou and colleagues introduced the concept of “structural clonotypes,” referring to groups of BCRs that recognize the same epitope due to high structural similarity among their antigen-binding sites (92). Schmidt and colleagues demonstrated that antibodies using different germline V genes converge to target the influenza hemagglutinin receptor-binding site (RBS) via a critical dipeptide motif in CDRH3 that mimics binding of RBS to sialic acid (93). This distinction highlights the importance of differentiating between “public antibodies”—those recognizing the same epitope due to high sequence similarity—and antibodies recognizing “public epitopes” that contain convergent binding features that can attract antibodies with lower sequence similarities (Fig. 1).

## PUBLIC ANTIBODIES AGAINST COMMON HUMAN PATHOGENS

**Fig 2:**
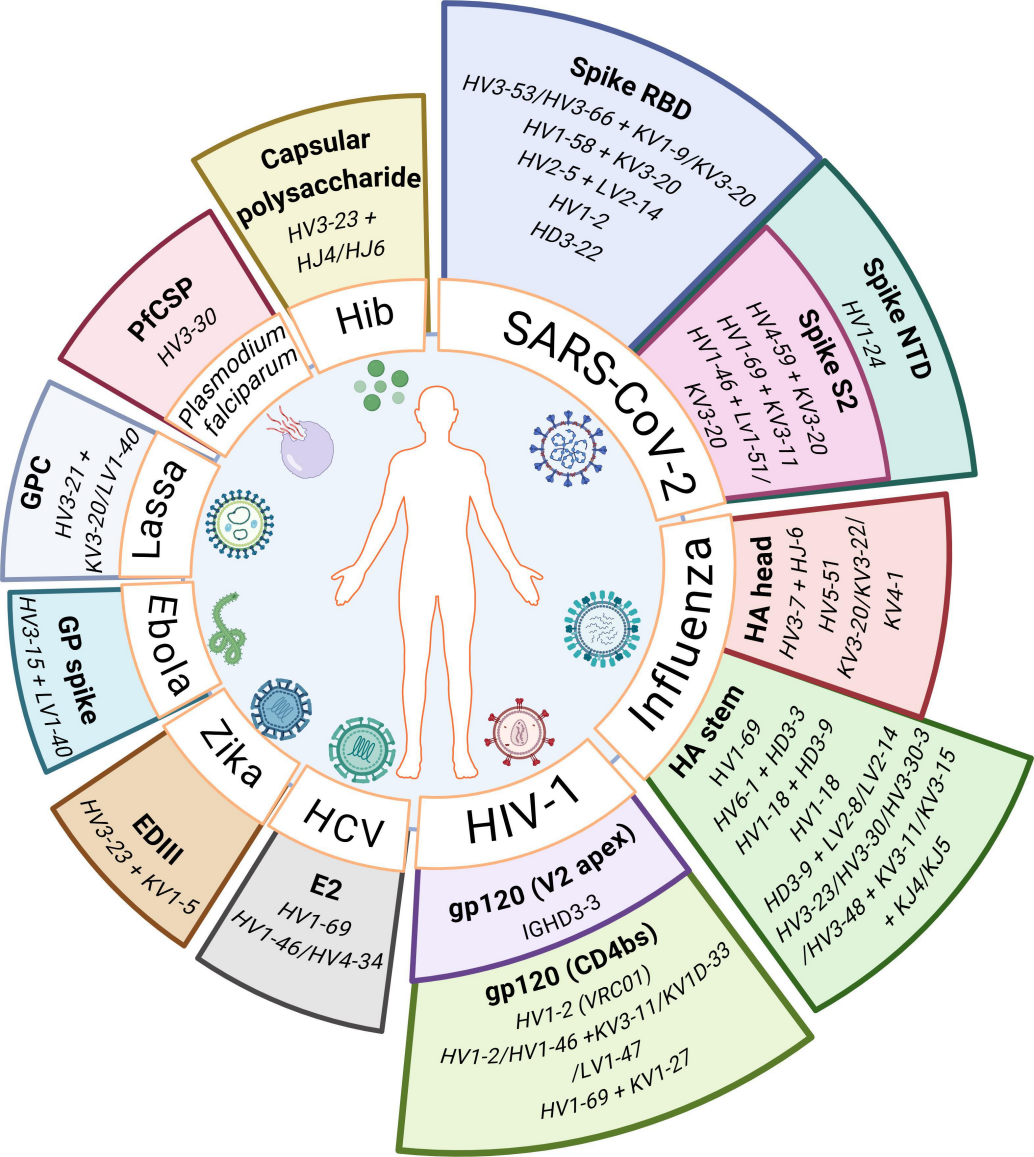
Public antibodies against common human pathogens. V**(**D)J gene usage in the heavy and/or light chains of reported public antibodies against commonly encountered pathogens. HV/HD/HJ, heavy chain; LV/LJ or KV/KJ, light chain; EDIII, envelope protein domain III; GP, glycoprotein; GPC, glycoprotein complex; PfCSP, *Plasmodium falciparum* circumsporozoite protein. For each pathogen, known public antibodies against various domains or epitopes in their proteins (listed in bold) are depicted.

The identification of human public antibodies elicited in response to specific pathogens is crucial to establish reliable signatures of pathogen-specific humoral immune responses (71). This section summarizes known public antibodies targeting commonly encountered human pathogens (Fig. 2).

### SARS-CoV-2

Multiple studies have identified public antibodies against SARS-CoV-2 in cohorts of COVID-19 infection and/or vaccination (63, 86, 94, 95). Two of the most common classes neutralize multiple variants by targeting spike’s RBD, and use *IGHV3-53* and *IGHV3-66* V genes, respectively (96, 97). The germlines of these genes differ in a single amino acid in FWR1 (74, 98), and they approach the RBD at a similar angle to directly compete with ACE2 binding, conferring neutralizing activity against SARS-CoV-2 (73). These two classes of public antibodies are associated with shorter CDRH3 lengths (74) and are predominantly paired with *IGKV1-9* or *IGKV3-20* (73). They have low somatic hypermutation rates, possibly because germline-encoded residues mediate the major contacts with the RBD (74). Somatic hypermutation in these antibodies has been associated with improved breadth of neutralization against emerging SARS-CoV-2 variants (78, 99).

Another widely documented class of cross-protective, RBD-targeting public antibodies use *IGHV1-58,* which is often paired with the *IGKV3-20* light chain. Its mode of recognition is driven by a configuration of predominantly germline-encoded aromatic residues that recognize phenylalanine 486 on the RBD (100–104). As this residue is part of a conserved RBD epitope, this class of public antibodies enabled cross-neutralization and protection against early SARS-CoV-2 variants of concern, such as Alpha (B.1.1.7) and Beta (B.1.351) (94, 105).

Yuan and colleagues reported public *IGHV2-5*/*IGLV2-14* antibodies with a CDRH3 length of 11 amino acids and a conserved motif targeting the RBD and cross-neutralizing a broad range of variants of concern (106). Interestingly, these antibodies had a strong preference for the IGHV2-5*02 allele, which possesses an aspartic acid at position 54 in the antigen binding site. Another class of RBD-targeting neutralizing public antibodies uses *IGHV1-2*, with germline-encoded residues in the heavy chain V gene interacting with RBD and an elongated CDRH3 enabling quaternary interactions with a neighboring RBD (97, 107). Finally, RBD-specific public antibodies defined by a YYDRxG motif encoded by *IGHD3-22* have been shown to cross-neutralize sarbecoviruses from SARS-CoV-1 to the BA.1 (Omicron) variant of SARS-CoV-2 (108).

Light chain-based convergence has also been observed in public antibodies targeting RBD. A class of *IGLV6-57* public antibodies has been shown to target a cryptic epitope on the RBD, facilitated by germline residues in the light chain CDRs, paired with diverse heavy chain V genes (63, 79). These genes converge through a common CDRH3 rearrangement. This class of antibodies likely contributed to immune pressure during the evolution of Omicron variants, as mutations within the Omicron RBD resulted in escape from recognition by these antibodies.

While the RBD harbors most of spike’s immunodominant epitopes, public antibodies targeting other regions have also been reported, including the NTD and S2 subunit. Interestingly, while most studies identified RBD-targeting B cells to be largely public, Voss and colleagues used proteomic sequencing to show that in the sera of four COVID-19-convalescent individuals, antibodies against the NTD were more prevalent than those against the RBD (57). This public anti-NTD response was dominated by *IGHV1-24* antibodies with variable light chains and CDRH3 lengths of 14–21 amino acids. This is consistent with other studies reporting the preferred usage of *IGHV1-24* among NTD-specific neutralizing antibodies with shorter CDRH3 regions (109–111) suggesting that antigen-binding sites targeting the NTD might reside predominantly outside CDR3 (63).

The S2 subunit is highly conserved across coronaviruses and elicits both neutralizing and non-neutralizing antibody responses (112–114). Strikingly, multiple studies looking for public antibodies among anti-spike B cell repertoires identified a pan-sarbecovirus public antibody targeting this subunit, which uses *IGHV4-59* and *IGKV3-20* and contains a short (6-amino acid) CDRH3 (63, 80, 89, 115, 116). We recently showed that affinity maturation of this class of public antibodies is driven by convergent and clonotype-enriched SHMs in the *IGKV3-20* light chain. Our data revealed that SHMs involved a polar to hydrophobic shift in contact residues that allows the antibody to access a cryptic central interface epitope that is exposed in the open conformation of S2 (80). Claireaux and colleagues identified a public antibody using *IGHV1-69*/*IGKV3-11* targeting the apex epitope on S2 that is exposed on the spike protein used in vaccines because of a conformational transition from the native pre-fusion state of spike (117), highlighting its potential as a COVID-19 vaccine-specific antibody signature. Multiple studies have identified a class of *IGHV1-46* broadly neutralizing antibodies (bnAbs) that recognize a highly conserved stem-helix epitope on S2 and can broadly neutralize betacoronaviruses (118–120). This class of public antibodies shows two modes of binding that differ in the approach angle of the heavy chain, depending on pairing with either the *IGLV1-51* or *IGKV3-20* light chains, both of which contribute germline-encoded contacts. Within the *IGKV3-20* binding mode, the interaction with the stem-helix epitope is further driven by a PPxF motif within a β-turn in the 10-amino-acid-long CDRL3 (119).

### Influenza

Conserved antibody signatures across different strains of influenza virus have been widely identified (71). Antibodies elicited by infection or vaccination primarily target the HA glycoprotein, which has a highly variable head domain that is immunodominant over its conserved stem region (66, 121). The head domain’s high variability limits the identification of public antibodies because of the spatiotemporal variability in exposure to circulating strains (122, 123). Despite this challenge, multiple studies have identified a public *IGHV3-7*/*IGHJ6* antibody with an 18-amino acid CDR3 that recognizes the Sa region of the HA head of the influenza A/California/04/09 H1N1 pandemic strain (64, 124–126). Using proteomic sequencing of serum antibodies, Adamson and colleagues also identified *IGHV3-7* and *IGHV5-51* as one of the most common V gene families elicited upon influenza A/(H1N1)pdm09 vaccination (58). They reported preferential usage of light chain genes *IGKV3-20*, *IGKV3-11*, and *IGKV4-1* among anti-H1 serum antibodies.

Unlike HA’s head, antibody responses against the stem tend to be more convergent, owing to its conserved nature across different strains of influenza A (127–130). One of the most well-known examples of a public antibody response is that of the *IGHV1-69* antibodies that target the conserved group 1 HA stem (71, 131–133). Their public nature is attributed to critical hydrophobic residues in the heavy chain CDR2 region that are sufficient to determine binding. This enables more diversity in the CDR3 and light chain pairings, thus allowing more sequences to be grouped under this public antibody cluster (71, 131, 132). Within the CDR2 of *IGHV1-69*, the phenylalanine (F) residue at position 54 is known to make a key contact with HA (133). A predicted 13% of the general population possess an allele of *IGHV1-69* that has a Leucine (L) instead of the phenylalanine at position 54, and interestingly, this substitution abolishes HA recognition across different HA stem-specific antibody clonotypes that use the *IGHV1-69* gene (134). This substitution has also been shown to influence the frequency of *IGHV1-69* antibodies in the antibody repertoire, a phenomenon that can be attributed to copy number variation, as well as differences in clonal expansion and somatic hypermutation (SHM) frequencies of *IGHV1-69* antibodies (69).

In addition to the *IGHV1-69* antibodies, Joyce and colleagues identified three multi-donor classes of public anti-HA stem antibodies with broad neutralizing capacity, using the *IGHV6-1*/*IGHD3-3*, *IGHV1-18*/*IGHD3-9,* and *IGHV1-18* genes with a Q-x-x-V motif between residues 98–100 in the heavy chain CDR3 (135). Among these, the *IGHV6-1* class elicits the broadest neutralization, against all influenza A subtypes (136, 137). Guthmiller and colleagues identified a class of bnAbs that target an anchor epitope on HA-stem (138). These antibodies use a restricted set of heavy (*IGHV3-23*, *IGHV3-30*, *IGHV3-30-3*, *IGHV3-48*) and light (*IGKV3-11*, *IGKV3-15* with *IGKJ4*, *IGKJ5*) chain V and J genes. The light chain CDR3s of this class of public antibodies are 10-amino-acids-long and possess a germline-encoded NWP motif, which in combination with Y58 in CDRH2 contribute to binding to the anchor epitope.

Another class of HA stem-specific public antibodies uses *IGHD3-9* and an 18-amino acid CDRH3, which are commonly paired with *IGLV2-8* and *IGLV2-14* light chains (70, 71, 139–141). The recurrent identification of public antibodies against the HA stem across different studies underscores their potential as signatures of the B cell repertoire elicited in response to influenza A exposure.

### Human immunodeficiency virus 1

One of the biggest challenges in the pursuit of combating human immunodeficiency virus-1 (HIV-1) is rapid intra-host viral evolution, which results in continual escape from neutralizing antibodies produced over the course of an active infection (142). This has made identifying public bnAbs difficult (143, 144). While most HIV-1 infections do not elicit bnAbs, the highly potent VRC01 class of public bnAbs has been widely detected in those that do (145–148). VRC01 bnAbs are extensively divergent in their heavy and light chain variable region sequences (50%–60% similarity) while retaining some convergent features, such as the inclusion of *IGHV1-2*, high somatic hypermutation rates, a five amino acid CDRL3 and a short and flexible CDRL1 (149–151). They neutralize HIV-1 by recognizing the CD4-binding site (CD4bs) in the gp120 subunit of the envelope glycoprotein (Env), preventing entry into host cells *via* CD4 (152). However, allelic variants of *IGHV1-2,* namely, IGHV1-2*04 and IGHV1-2*05, are unsuccessful in developing into VRC01-like BCRs. While this has been attributed to a tryptophan to arginine substitution at position 50 in case of IGHV1-2*05, it is currently unclear why the IGHV2*04 allele cannot generate VRC01-like antibodies (81, 82, 85).

Scheid and colleagues identified a group of highly active agonistic CD4bs antibodies containing *IGHV1-2* or *IGHV1-46* heavy chains and *IGKV3-11/IGKV1D-33/IGLV1-47*, which recognize an overlapping neutralizing epitope to that of the VRC01 class, with 10 shared contacts (153). Another class of neutralizing antibodies in HIV-1-infected donors targets the gp120 CD4bs, using *IGHV1-69* and *IGKV1-27* and displays high CDRH3 and CDRL3 similarity and convergent somatic hypermutation in *IGHV1-69* (87, 88).

Another common target of HIV-1 bnAbs is the V2 apex on gp120 (154). A class of public antibodies that target a glycan-strand C core epitope within the V2 apex has been shown to rely on a highly conserved, germline-encoded anionic YYD motif within the *IGHD3-3* gene (155). Upon sulfation, this motif forms a stable complex with the V2 apex epitope (156). These antibodies use different V genes although with high (>99%) sequence similarity and are capable of neutralizing multiple HIV-1 isolates in their unmutated germline versions (155).

### Other viruses

Public antibodies against flaviviruses such as hepatitis C virus (HCV), dengue virus, and Zika virus have also been reported. Like HIV-1, HCV causes chronic infection in humans with the evolution of intra-host viral variants, motivating efforts to identify and elicit bnAbs (157, 158). Similar to influenza A and SARS-CoV-2, the most potent public bnAbs against HCV contain *IGHV1-69*, highlighting the versatility of this V gene in impacting antibody functionality (132, 159, 160). These anti-HCV public antibodies target antigenic region 3 (AR3) of the E2 protein, which interacts with the CD81-binding site on host cells (161–167) and is associated with a highly hydrophobic CDRH2 and an intramolecular disulfide bond within CDRH3, with most interactions mediated by the heavy chain (168, 169). Ogega and colleagues identified non-*IGHV1-69* public bnAbs primarily containing *IGHV1-46* and *IGHV4-34* that target three other neutralizing epitopes on HCV’s E2 protein (the highly conserved front-layer FRLY epitope, the β-sandwich epitope, and back layer epitope) (170).

Parameswaran and colleagues looked for convergent signatures in the antibody repertoires of 44 dengue-infected individuals and identified 10-mer and 13-mer public CDR3 sequences associated with different V and J genes (171). Robbiani and colleagues identified a class of potent *IGHV3-23*/*IGKV1-5* public neutralizing antibodies against Zika virus in six individuals with high neutralizing antibody titers (172). These antibodies bind to the lateral ridge of envelope protein domain III, which mediates host cell attachment. They also cross-neutralize the DENV1 serotype of dengue virus.

Although several limitations prevent access to human samples from rarer infections, multiple studies have identified a class of *IGHV3-15*/I*GLV1-40* public antibodies against Ebola virus upon infection or vaccination (173–177). These antibodies target a highly conserved region in the receptor-binding site of the glycoprotein spike and confer neutralizing activity (173). Hastie and colleagues identified a class of public anti-Lassa virus antibodies using *IGHV3-21* paired with *IGKV3-20* or *IGLV2-14*. These antibodies target its glycoprotein complex and achieve pan-Lassa virus neutralization (178).

### Protozoa

Pieper and colleagues identified a rare class of public antibodies binding to the surface of erythrocytes infected with *Plasmodium falciparum*, the protozoan that causes malaria (179). These antibodies contained an insertion encoding a collagen-binding inhibitory receptor (leukocyte-associated immunoglobulin-like receptor 1) between the VH and CH1 domains. This insertion, which presumably occurs during the process of class-switch recombination, was observed in 5%–10% of malaria-infected individuals in a cohort in Mali and Tanzania. Tan and colleagues identified a vaccine-induced class of *IGHV3-30* public antibodies displaying extensive somatic hypermutation that possess dual specificity toward the NANP repeats and N-terminal junction of *P. falciparum’s* circumsporozoite protein and are more potent than antibodies that recognize only one of these regions (83). This study also highlighted a novel variant of *IGHV3-30* that encodes a tryptophan instead of a serine at position 52. The tryptophan at position 52 (W52) has been shown to be a key residue for the recognition of the linear NANP repeat epitope that is targeted by another class of *IGHV3-33/IGKV1-5* public antibodies that also express W52 in the *IGHV3-33* germline (180).

### Bacteria

Multiple studies have reported a class of public antibodies using *IGHV3-23* and *IGHJ4* or *IGHJ6* with an invariable GYGF/MD CDRH3 sequence motif and Vκ2 light chain gene that target the capsular polysaccharide of *Haemophilus influenzae* type b (Hib) (181–187) which can cause life-threatening illness in young children. In addition, Trück and colleagues identified Hib-specific public 10-amino acid CDRH3 sequences in five participants upon Hib-Meningococcus C polysaccharide-protein conjugate vaccination and revealed that the number of Hib-specific CDR3 sequences correlated with the antibodies’ avidity for Hib (187).

## EVOLUTIONARY SIGNIFICANCE AND APPLICABILITY OF PUBLIC ANTIBODIES

The remarkable diversity of the antibody repertoire raises questions about the evolutionary significance of public antibody responses. Dunand and Wilson attributed the convergence of *IGHV1-69* anti-HA stem-specific responses upon influenza virus infection or vaccination to the highly conserved nature of the HA stem region (188). Upon repeated exposure to different antigenic variants of HA, pre-existing memory B cells using *IGHV1-69* would preferentially be recalled, leading to a robust amplification of these antibodies. This repeated exposure enhances the convergence of *IGHV1-69* antibodies within human populations. This concept of “antibody focusing” was further reinforced by the prevalence of *IGHV1-6*9 antibodies observed following sequential immunization of humanized mice (transgenic mice expressing human antibody repertoires) with a group 1 HA-stalk-based nanoparticle (189). A comparable phenomenon has been noted in the context of SARS-CoV-2, where Omicron breakthrough infections have been shown to promote the convergent restoration of *IGHV3-53/66* bnAbs, which were primarily triggered by prior exposure to the original Wuhan-Hu-1 strain, along with the accumulation of SHMs following the breakthrough infection (190). These findings suggest that repeated exposure to antigenically similar strains of a pathogen, whether through infection or vaccination, can shape the antibody repertoire to preferentially generate highly similar antibodies targeting immunodominant epitopes, thereby contributing to their public nature. While the reasons behind the widespread use of antibody genes like *IGHV1-69* against diverse antigens remain unclear, one plausible explanation is the existence of certain germline-encoded paratope features which facilitate interactions with different epitopes. In line with this, Sangesland and Lingwood proposed that public antibodies and BCRs can be considered “innate-like” immune receptors, as they contain germline-encoded motifs capable of recognizing epitopes from diverse pathogens (191). They suggest that, during the evolution of antigen receptors, certain antibody genes were initially selected for their ability to recognize specific antigens encountered by the host, and multiple “unanticipated” specificities of these antibodies to different epitopes evolved as “spandrels” or traits that were coupled with the primary epitope specificity. For instance, the same group demonstrated that the IGHV1-2*01 allele, used by the VRC01-class of bnAbs, can also recognize the sacrolipid core of lipopolysaccharides (LPS) on the surface of gram-negative bacteria (192). This framework offers an explanation for the versatility of germline genes such as *IGHV1-69*, which has evolved to recognize a broad range of pathogens.

Similarly, Shrock and colleagues identified germline-encoded amino acid binding (GRAB) motifs in 18 V gene segments that specifically bind to selected amino acids on linear public epitopes from different human viral pathogens (193). They emphasized that such GRAB motifs capable of recurrently recognizing public epitopes across pathogens form the basis for the repeated selection of antibodies harboring these motifs in response to various infectious threats. Collectively, these theories, supported by experimental data, suggest that the convergence of antibody responses likely evolved as a result of selective pressures favoring antibodies with motifs capable of effectively binding to antigens from diverse pathogens encountered over the course of evolution of the adaptive immune system.

The phenomenon of recurrent selection of B cells that produce highly potent broadly neutralizing antibodies has paved the way to a new technology for vaccine design termed reverse vaccinology 2.0, where immunogens are designed to target specific germline B cells (194, 195). The goal is to selectively activate germline B cells that can develop into mature B cells expressing the desired bnAbs. This technology has been employed to design vaccines against highly mutable pathogens like HIV-1 and influenza virus with an aim of inducing bnAbs (134, 196–199). In order to design germline-targeting immunogens, the germline B cell is required to have detectable affinity in its unmutated state, against the cognate immunogen (195, 200). This poses a big challenge in optimizing the choice of immunogens that should be able to both recurrently activate germline B cells in different individuals and target germline B cells that are frequent in the human B cell repertoire. This often demands further optimization of the native immunogen present in the pathogen. For example, the native gp120 glycoprotein of HIV-1 was unsuccessful in eliciting the VRC01-class of bnAbs due to which a highly mutated optimized version of the outer domain of gp120, namely eOD-GT6, had to be designed to target germline VRC01-like B cells (146, 153, 197). A further optimized version of this immunogen, named eOD-GT8, elicited VRC01-like germline B cells in 97% of the individuals immunized (198, 201). Similarly, in case of influenza, the *IGHV1-69* class of HA stem-specific bnAbs has been a popular target of germline-targeting vaccines (189, 202). While the germline *IGHV1-69* bnAbs themselves are unable to bind HA in their soluble form, the corresponding BCRs on the cell surface are able to bind HA, thus triggering the activation of the desired germline B cells (134, 203). However, subtype bias resulting from previous infection with a particular strain in what has been traditionally referred to as original antigenic sin (OAS) or immune imprinting has limited the effectiveness of such germline-targeting vaccines in activating the desired germline B cells in case of influenza (204, 205). Nonetheless, the phenomenon of antibody convergence can be effectively leveraged to design germline-targeting vaccines that specifically induce broadly neutralizing antibodies of interest.

Convergent antigen-antibody interactions across human populations may also play a critical role in host-pathogen co-evolution. Public antibodies, by targeting immunodominant public epitopes, can impose selective pressures on pathogens, potentially driving the emergence of escape mutations (193, 206). This can be countered by the host on a short time scale (*via* somatic hypermutation, leading to antibodies that recognize the escape mutations) or on a longer time scale (by accumulating allelic polymorphisms in the V genes) (78, 106, 207). Thus, identifying public antibodies could be crucial for predicting escape mutations in emerging viruses, enhancing our pandemic preparedness. Public antibodies can also be used as tools to reconstruct affinity maturation pathways using antibody sequences from multiple individuals and identify key mutations that contribute to increased antibody affinity and functionality (63, 80, 94). This can further inform the design of immunogens that effectively elicit high-affinity antibodies.

Therefore, the continued identification and characterization of public antibodies will improve our understanding of humoral immunity and our clinical defenses against various pathogens threatening human health.
